# Commentary: Hepatitis B virus infection: an insight into the clinical connection and molecular interaction between hepatitis B virus and host extrahepatic cancer risk

**DOI:** 10.3389/fimmu.2023.1200405

**Published:** 2023-05-17

**Authors:** Sergey Morozov, Sergey Batskikh

**Affiliations:** ^1^ Department of Gastroenterology, Hepatology and Nutrition, Federal Research Center of Nutrition and Biotechnology, Moscow, Russia; ^2^ Department of Hepatology, Moscow Clinical Scientific Center n.a. A.S. Loginov, Moscow, Russia

**Keywords:** previous hepatitis B virus infection, occult hepatitis B, extrahepatic cancer, risk factor, hepatitis B virus-related non-liver malignancy

## 1 Introduction

In a recently published paper, Min et al. (1) reviewed available data on the relationship of hepatitis B virus (HBV) infection and non-liver cancer risk. We agree with the authors that the role of HBV in extrahepatic malignancies is underestimated and would like to address some details that were not directly mentioned in the paper.

## 2 Variability of hepatitis B virus-related malignancy risk values and possible reasons

Even within one type of cancer, there is a large heterogeneity of risks associated with HBV infection. For example, in pancreatic cancer, they range from 0.89 to 4.0 (2, 3). This may be caused by the methodology used in the studies. In most of them, the control group included subjects with a negative blood test on hepatitis B surface antigen (HBsAg). Such an approach could result in the enrollment of patients with previous exposure to HBV infection to the “HBV-free” group and make the analyzed risks lower. This is especially important, as the majority of data were received from populations with a high prevalence of HBV infection, where chances to obtain false-negative results (type II error) were greater. Of note, when previous HBV infection was taken into account, the authors revealed 2–4 times greater odds of HBV-related malignancies (3, 4).

HBV infection is a dynamic process that has several phases. During the final (V) phase, HBsAg is cleared out of the blood (HBsAg-negative phase) and the infection may remain further in the following forms: as a low-level viral replication (“occult HBV infection”) and/or an integration of the viral DNA to the host genome (5, 6).

We agree with the authors of the review that the integration of the HBV DNA and the production of the HBx protein are the main factors that provoke an intrahepatic immune imbalance and that they are the key factors of liver carcinogenesis. Integration of the virus in specific regions of the host’s genome may lead to the initiation of insertional mutagenesis, induction of chromosomal instability of key genes, and the generation of a permanent source of viral proteins (especially HBx), that can induce cancer development (5, 7). These pathogenetic mechanisms seem to be universal and actual not only for liver cancer but also for non-liver HBV-associated malignancy. However, a major factor that plays a crucial role in the development of any HBV-associated tumor is time, as cancer usually develops several decades after integration of the virus, and this process probably depends on the duration of exposure to the products of the expression of viral genes (8).

## 3 Discussion

Based on the abovementioned analysis, we would like to summarize the unmet needs and the overview of possible solutions.

First of all, the growing evidence of the involvement of HBV infection to non-liver outcomes (triggering the autoimmune disorders and causing non-liver cancer risks) requires broader awareness by all groups of medical specialists (9).

Second, for better understanding of the role of HBV infection in the development of non-liver malignancies, we need more well-planned studies that compare not only HBsAg-positive and HBsAg-negative groups of subjects but also those with previous exposure to HBV infection (anti-HBc-positive) and without it (anti-HBc-negative).

Up to this moment, only a few studies have provided direct molecular and genetic evidence on the involvement of HBV infection in non-liver cancer development (4, 10, 11). For example, in pancreatic cancer, our data revealed HBx protein expression in the pancreatic tumor tissue in four out of five HBsAg-negative and anti-HBc-positive patients with pancreatic ductal adenocarcinoma (4). In three of them, the replicative competence of HBV was confirmed by the detection of covalently closed circular DNA (cccDNA). In one patient, the expression of HBx was caused only by the integration of the HBV into the genome of pancreatic cells. These fragments of viral DNA, which preserve the open reading frame and express HBx, may serve as a basis for carcinogenesis.

Moreover, the authors of the review mentioned the need for targeted screening for established HBV-related cancers in populations of subjects with chronic HBV infection (1). This approach might be promising, but it does not involve subjects with previous HBV infection who have even greater risks of cancer development. We suppose that as a first step, wider anti-HBc screening programs, at least in regions with a high prevalence of HBV infection, may help detect greater numbers of patients who need further surveillance programs.

Finally, the development of novel treatment options for HBV infection that can result to a sterilizing cure instead of a functional cure is urgently needed to minimize the risks of HBV-related malignancies.

## Author contributions

All authors listed have made a substantial, direct, and intellectual contribution to the work and approved it for publication.
